# Inclination Effect on the Periodic Response of a Symmetrical MEMS Gyroscope

**DOI:** 10.3390/mi13101569

**Published:** 2022-09-21

**Authors:** Lijuan Zhang, Huabiao Zhang, Xinye Li, Yunxiao Ji

**Affiliations:** 1 School of Automobile and Transportation, Tianjin University of Technology and Education, Tianjin 300222, China; 2 School of Mechanical Engineering, Tianjin University of Commerce, Tianjin 300134, China; 3 School of Mechanical Engineering, Hebei University of Technology, Tianjin 300401, China; 4 Tianjin Juxinhongtai Metal Material Company Limited, Tianjin 300112, China

**Keywords:** inclination angle, symmetrical MEMS gyroscope, periodic response, bifurcation, the singularity theory

## Abstract

The inclination effect caused by fabrication errors on the periodic response of a symmetric MEMS gyroscope is investigated. The dynamic equation is established considering the inclination effect on support stiffness and electrostatic forces. The periodic response is obtained by the averaging method. The two-variable singularity theory is employed to study the bifurcation characteristics and give transition sets on the DC-AC voltage plane, which divide the plane into four persistent regions. The amplitude-frequency curves demonstrate that only the two persistent regions with low voltages are feasible for the gyroscope. Both over-etching and under-etching reduce the feasible region. The effect of parameters on the performance is present. The mechanical sensitivity and nonlinearity increase with the voltages. With the increase in the inclination angle, the mechanical sensitivity and nonlinearity decrease first and then increase. The full temperature stability of the mechanical sensitivity is also considered. The variation in mechanical sensitivity with temperature is small at a large voltage and negative inclination angle. Under-etching, which leads to small nonlinearity and good temperature stability, is more beneficial to the performance of the gyroscope than over-etching.

## 1. Introduction

MEMS gyroscopes are widely used for their small size, light weight, low manufacturing cost, and high reliability [1]. However, nonlinearities, including nonlinear stiffness and nonlinear electrostatic force, usually exist in MEMS gyroscopes. Nonlinearities can lead to multi-solutions and softening/hardening characteristics of the response, which affect the stability, sensitivity, and reliability of MEMS gyroscopes. Therefore, the nonlinear dynamics of MEMS gyroscopes have attracted significant attention from researchers.

Braghin et al. [2] obtained a high deformation range of the support beams of a tuning-fork MEMS gyroscope and investigated the nonlinear vibrations both experimentally and numerically. Tsai et al. [3] studied the stability and resonance of a micro-machined gyroscope considering the nonlinearities resulting from flexure springs and electrostatic force. The external resonance and non-resonant hard excitation were considered. Martynenko et al. [4] investigated the nonlinear dynamics of a MEMS tuning fork gyroscope with elastic rods on the moving base using the asymptotic two-scales method. Lajimi et al. [5] examined the effect of the DC bias voltages, the AC drive voltage, drive frequency, and the quality factors on the nonlinear dynamics and the sensitivity of a beam-rigid body gyroscope based on a computational shooting method. Shang et al. [6] demonstrated the nonlinear vibration of a parametric exciting gyroscope considering the principal parameter resonance and 1:1 internal resonance. Hao et al. [7] discussed the influence of DC bias voltage and comb spacing on the nonlinearity of electrostatic force and the dynamic response of a 4-DOF electrostatically driven gyroscope. Liang et al. [8] analyzed the nonlinear mechanism of MEMS vibratory ring gyroscopes in which the gyroscopic coupling and geometrically and structurally nonlinear couplings are all considered. Zhang et al. [9] investigated the effect of driving and sensing voltages on the bifurcation of the periodic response of a micro gyroscope with comb driving and parallel-plate sensing.

Currently, most MEMS gyroscopes are fabricated by the deep reactive ion etching (DRIE) process. Inevitably, over-etching or under-etching will lead to the non-parallelism of the working planes of the driving and sensing combs, as well as the change in the cross-section of the support beams, which will affect the performance of the gyroscope.

To clarify the influence of fabricating error, Tay et al. [10] investigated the effect of non-parallel plates on the capacitance, sensitivity, electrostatic force, and electrostatic spring constant of a micro accelerometer using a theoretical approach and finite-element analyses. Dong et al. [11] considered the effect of non-parallel combs on the reliable operation range for step and pulse signals in different capacitive configurations and gave the analytical expressions of the critical step acceleration and the critical time duration of the pulse acceleration of a capacitive sensor whose comb electrodes had a small angle of the decline. Guo et al. [12] introduced a new theoretical model of plate capacitor for a MEMS comb actuator. The bulk fabrication process and edge effect on the capacitance are both considered using the integration method and conformal transformation theory. Zhong et al. [13] studied the inclination effect of the fingers on the capacitance, driving electrostatic force, and electrostatic spring constant and discussed the nonlinear dynamics of a MEMS resonator. Yan et al. [14] analyzed the electrostatic tuning mechanism of electrical stiffness produced by the electrostatic force and gave the dependence of resonance frequency variation on the inclination angle and DC bias voltage of an inclined disk resonator array. Zhang et al. [15] studied the influence of inclination angle on the resonance frequency, stiffness hardening effect, and sensitivity of a 4-DOF MEMS gyroscope.

However, existing studies have mainly focused on the influence of the inclination angle on the electrostatic force. For actual gyroscopes, the inclination angle can also vary the cross-section of the support beams, thereby changing the support stiffness and resonant frequency.

In this paper, we propose an investigation of the nonlinear dynamics of a symmetrical MEMS gyroscope considering the inclination effect on both the electrostatic force and support stiffness of the gyroscope. The structure of the paper is organized as follows. In Section 2, the dynamical model of the MEMS gyroscope is established considering the influence of the inclination angle. In Section 3, the periodic response is obtained based on the averaging method. In Section 4, we study the effect of parameters on the bifurcation characteristics, amplitude–frequency response, and the performance of the gyroscope. Finally, the conclusions are summarized.

## 2. The Dynamical Model of the MEMS Gyroscope Considering the Influence of the Inclination Angle

### 2.1. The Dynamical Model of the MEMS Gyroscope

**Figure 1:**
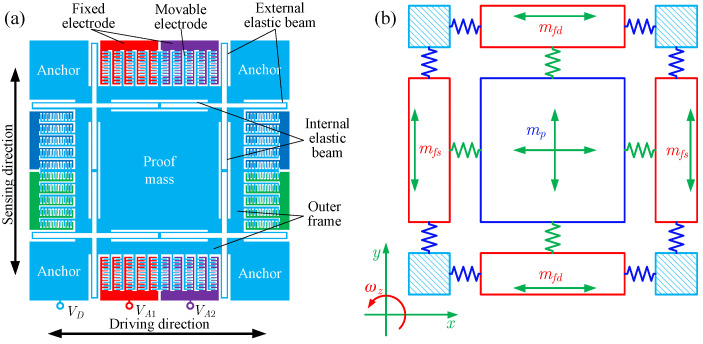
The schematic diagram of the symmetrical MEMS gyroscope and the corresponding spring-mass model, (**a**) schematic diagram of the symmetrical MEMS gyroscope [16], and (**b**) corresponding spring-mass model.

Figure 1a is a schematic diagram of the symmetrical gyroscope [16]. The proof mass is connected to the outer frame by internal elastic beams and then supported on the anchor by external elastic beams. Figure 1b shows the corresponding spring-mass model. The blue and green springs represent the external and internal elastic beams. *x* and *y* are the driving and sensing directions, and *z* is the direction of the vertical paper facing outward. $m_{p}$ is the proof mass, $m_{fd}$ and $m_{fs}$ are the masses of the driving and sensing outer frames, and $m_{fd}=m_{fs}=m_{i}$. The proof mass can move in two directions, while the driving and sensing outer frame can move in only one direction. During the gyroscope operation, the DC voltage $V_{D}$ is applied to the movable structure, and the AC voltages $V_{A1}$ and $V_{A2}$ are applied to the fixed combs in the driving direction. The electrostatic forces drive the proof mass and the driving outer frame to vibrate in the driving direction. When the carrier of the gyroscope has an angular velocity around the z direction, the proof mass vibrates in the sensing direction due to the Coriolis effect. Then the vibration is transmitted to the sensing outer frame. The angular velocity can be determined by measuring the vibration in the sensing direction.

**Figure 2:**
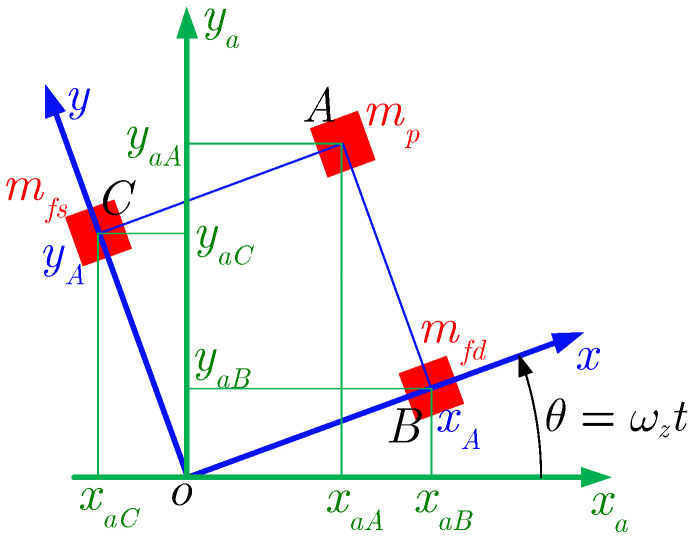
The relation between the displacements of the gyroscope coordinate system and the inertial coordinate system.

Figure 2 shows the relation between the displacement of the gyroscope coordinate system and the inertial coordinate system, where x-o-y is the gyroscope coordinate system and $x_{a}\text{-}o\text{-}y_{a}$ is the inertial coordinate system. The displacement of the proof mass and the driving and sensing frames in the inertial coordinate system can be written as

(1) $$\begin{array}{c} x_{aA}=x_{A}\cos\omega_{z}t-y_{A}\sin\omega_{z}t,y_{aA}=x_{A}\sin\omega_{z}t+y_{A}\cos\omega_{z}t,\\ x_{aB}=x_{A}\cos\omega_{z}t,y_{aB}=x_{A}\sin\omega_{z}t,\\ x_{aC}=-y_{A}\sin\omega_{z}t,y_{aC}=y_{A}\cos\omega_{z}t \end{array}$$

The velocities can be obtained by the derivative of Equation (1) to time *t*.

(2) $$\begin{array}{c} {\dot{x}}_{aA}=\left({\dot{x}}_{A}-y_{A}\omega_{z}\right)\cos\omega_{z}t-\left(x_{A}\omega_{z}+{\dot{y}}_{A}\right)\sin\omega_{z}t,\\ {\dot{y}}_{aA}=\left({\dot{x}}_{A}-y_{A}\omega_{z}\right)\sin\omega_{z}t+\left(x_{A}\omega_{z}+{\dot{y}}_{A}\right)\cos\omega_{z}t,\\ {\dot{x}}_{aB}={\dot{x}}_{A}\cos\omega_{z}t-x_{A}\omega_{z}\sin\omega_{z}t,\\ {\dot{y}}_{aB}={\dot{x}}_{A}\sin\omega_{z}t+x_{A}\omega_{z}\cos\omega_{z}t,\\ {\dot{x}}_{aC}=-y_{A}\omega_{z}\cos\omega_{z}t-{\dot{y}}_{A}\sin\omega_{z}t,\\ {\dot{y}}_{aC}=-y_{A}\omega_{z}\sin\omega_{z}t+{\dot{y}}_{A}\cos\omega_{z}t \end{array}$$

Thus, the kinetic energy of the system is

(3) $$T=\frac{1}{2}m_{p}\left({\dot{x}}_{aA}^{2}+{\dot{y}}_{aA}^{2}\right)+m_{i}\left({\dot{x}}_{aB}^{2}+{\dot{y}}_{aB}^{2}+{\dot{x}}_{aC}^{2}+{\dot{y}}_{aC}^{2}\right)$$

We can also give the potential energy and Rayleigh dissipation function as

(4) $$V=\left(2k_{o}+k_{i}\right){x_{A}}^{2}+\left(\alpha_{o}+\frac{1}{2}\alpha_{i}\right){x_{A}}^{4}+\left(2k_{o}+k_{i}\right){y_{A}}^{2}+\left(\alpha_{o}+\frac{1}{2}\alpha_{i}\right){y_{A}}^{4}$$

(5) $$D=\frac{1}{2}c\left({\dot{x}}_{A}^{2}+{\dot{y}}_{A}^{2}\right)$$

where $k_{o},k_{i},\alpha_{o},\mathrm{and}\;\alpha_{i}$ are the linear and nonlinear stiffness of the external and internal support beams and *c* is the damping coefficient. According to the Lagrange equation, the dynamic equation of the system can be obtained as follows:

(6) $$\begin{array}{c} \left(m_{p}+2m_{i}\right)\ddot{x}-2m_{p}\omega_{z}\dot{y}+\left[4k_{o}+2k_{i}-\left(m_{p}+2m_{i}\right){\omega_{z}}^{2}\right]x+\left(4\alpha_{o}+2\alpha_{i}\right)x^{3}+c_{x}\dot{x}=F_{ex}\\ \left(m_{p}+2m_{i}\right)\ddot{y}+2m_{p}\omega_{z}\dot{x}+\left[4k_{o}+2k_{i}-\left(m_{p}+2m_{i}\right){\omega_{z}}^{2}\right]y+\left(4\alpha_{o}+2\alpha_{i}\right)y^{3}+c_{y}\dot{y}=F_{ey} \end{array}$$

Considering that $\left(m_{p}+2m_{i}\right){\omega_{z}}^{2}\ll 4k_{o}+2k_{i},\dot{y}\ll \dot{x}$, Equation (6) can be simplified as

(7) $$\begin{array}{c} M\ddot{x}+Kx+\Gamma x^{3}+c\dot{x}=F_{ex}\\ M\ddot{y}+2m_{p}\omega_{z}\dot{x}+Ky+\Gamma y^{3}+c\dot{y}=F_{ey} \end{array}$$

where

(8) $$M=m_{p}+2m_{i},K=4k_{o}+2k_{i},\Gamma =4\alpha_{o}+2\alpha_{i}$$

where $F_{ex}\mathrm{and}\;F_{ey}$ denote the electrostatic forces in the driving and sensing directions, respectively. Their expressions will be given below.

### 2.2. The Electrostatic Forces in the Driving and Sensing Directions with Inclination Effect

**Figure 3:**
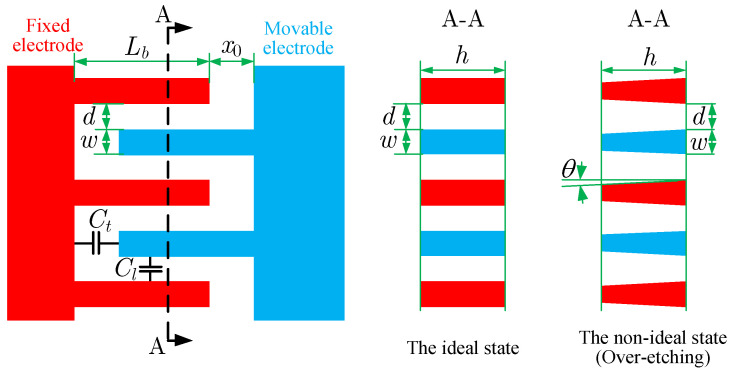
The capacitances and the inclination angle of the combs, where θ>0 denotes the over-etching state and θ>0 denotes the insufficient-etching state.

As shown in Figure 3, two kinds of capacitances exist between the combs, the longitudinal capacitance $C_{L}$ and the transverse capacitance $C_{T}$. For the non-ideal state, the working faces of the combs are not parallel, which consequently changes the capacitances. Assuming the inclination angle is small, the longitudinal capacitance considering the edge effect and the inclination angle is [12]

(9) $$\begin{array}{c} C_{L}=\frac{\varepsilon Nhl}{h\theta +d}+\frac{\varepsilon Nh}{\pi }\left\{1+\ln\left[1+\frac{\pi l}{h\theta +d}+\ln\left(1+\frac{\pi l}{h\theta +d}\right)\right]\right\}\\ +\frac{\varepsilon Nl}{\pi }\left\{1+\ln\left[1+\frac{\pi h}{h\theta +d}+\ln\left(1+\frac{\pi h}{h\theta +d}\right)\right]\right\} \end{array}$$

where θ is the inclination angle, *l* is the overlap length of combs, *h* is the structural thickness of the gyroscope, *d* is the spacing between the fingers, ε is the dielectric constant, and *N* is the number of fingers on a single side of the combs. Since the separation between the combs is large, the inclination effect on the transverse capacitance can be ignored. The transverse capacitance is given as

(10) $$C_{T}=\frac{\varepsilon Nh(w-h\theta )}{x_{0}}$$

where *w* is the finger’s width, and $x_{0}$ is the separation between the combs. According to Ref. [17], the electrostatic force can be obtained by

(11) $$F_{e}=\frac{1}{2}V^{2}\frac{\partial C}{\partial x}$$

where *C* and *V* are the capacitance and voltage between the electrodes, respectively. Thus, the electrostatic forces in the driving and sensing direction can be given as

(12) $$\begin{array}{c} F_{ex}=\frac{1}{2}{\left(V_{D}+V_{A}\cos\omega t\right)}^{2}\frac{\partial C_{r}}{\partial x}-\frac{1}{2}{\left(V_{D}-V_{A}\cos\omega t\right)}^{2}\frac{\partial C_{l}}{\partial x}\\ =4c_{4}\beta \frac{N\eta {V_{D}}^{2}}{2}\cos\omega \;t+\frac{N\eta {V_{D}}^{2}}{2}\left(f\left(x\right){\left(1+\beta \cos\omega \;t\right)}^{2}-\;f\left(-x\right){\left(1-\beta \cos\omega \;t\right)}^{2}\right)\;\\ +c_{3}\frac{N\eta {V_{D}}^{2}}{2}\left(\frac{{\left(1+\beta \cos\omega \;t\right)}^{2}}{{\left(x_{0}-x\right)}^{2}}-\frac{{\left(1-\beta \cos\omega \;t\right)}^{2}}{{\left(x_{0}+x\right)}^{2}}\right)\\ F_{ey}=\frac{1}{2}{V_{D}}^{2}\frac{\partial C_{t}}{\partial x}-\frac{1}{2}{V_{D}}^{2}\frac{\partial C_{b}}{\partial x}\\ =\frac{N\eta {V_{D}}^{2}}{2}\left(f(y)-\;f(-y)\right)\;+c_{3}\frac{N\eta {V_{D}}^{2}}{2}\left(\frac{1}{{\left(x_{0}-y\right)}^{2}}-\frac{1}{{\left(x_{0}+y\right)}^{2}}\right) \end{array}$$

where $C_{l}$ and $C_{r}$ are the capacitances on the left and right as the movable structure has a displacement *x*. $C_{t}$ and $C_{b}$ are the capacitances on the top and bottom with the displacement *y*.

(13) $$\begin{array}{c} c_{1}=\frac{h}{h\theta +d},c_{2}=\frac{\pi }{h\theta +d},c_{3}=\;h\left(w-h\theta \right),c_{4}=c_{1}+\frac{1}{\pi }\left(1+\ln\left(1+c_{1}\pi +\ln\left(1+c_{1}\pi \right)\right)\right),\\ f\left(x\right)=\frac{c_{1}\left(2+c_{2}\left(l+x\right)\right)}{\left(1+c_{2}\;\left(l+x\right)+\ln\left(1+c_{2}\left(l+x\right)\right)\right)\left(1+c_{2}\left(l+x\right)\right)} \end{array}$$

### 2.3. The Inclination Effect on the Restoring Force

**Figure 4:**
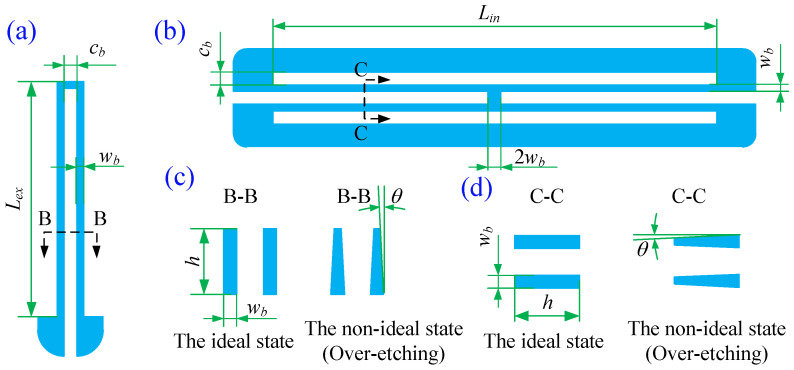
The structure of the support beams and the inclination angles, (**a**) the enlargement of the external elastic beam, (**b**) the enlargement of the internal elastic beam, (**c**) the enlargement of the section view B, and (**d**) the enlargement of the section view C, where $L_{ex}=800\;\mu m,L_{in}=1660\;\mu m,$ $c_{b}=30\;\mu m$, and $w_{b}=15\;\mu m$.

**Figure 5:**
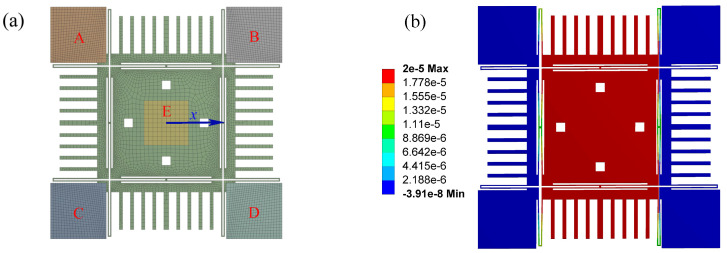
The finite element analysis of the restoring force (the displacement in the driving direction), where the displacement of the proof mass is x=20μm, T=300K,θ=0, and $F_{re}=7.5151\times 10^{-3}\;N$. (**a**) The FEA model, (**b**) The displacement contour.

The inclination angle also changes the cross-section of the support beams and the stiffness of the gyroscope, as shown in Figure 4. However, it is difficult to represent the inclination effect on the stiffness analytically. Thus, a finite element analysis is carried out. We also consider the effect of the environmental temperature on Young’s modulus of the material.

(14) $$E=E_{0}+\upsilon E_{0}\left(T-T_{0}\right)$$

where $T_{0}=300\;K$, $E_{0}=170\;\mathrm{GPa}$ is Young’s modulus of monocrystalline silicon at 300 K, and $\upsilon =5\times 10^{-5}$. The FEA model is presented in Figure 5. The combs are ignored since they do not affect the calculation results of the restoring force. The locally refined hexahedron mesh generated by the sweep method is used. The minimum element edge length is 6.21 μm, and the total number of elements is 15,956. The structures of the driving and sensing directions are wholly symmetrical, so only the restoring force of the *x* direction is considered. The fixed constraint is applied to the surface A, B, C, and D. The environmental temperature is applied to the structure as a thermal load. By giving a displacement in the *x* direction at the center E of the proof mass, the restoring force of the fixed constraint can be obtained by the software ANSYS Workbench 14.5.

**Figure 6:**
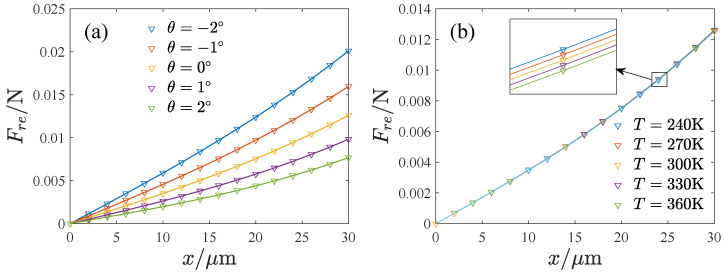
The relation between the displacement and the restoring force, where the symbols denote the FEA results and the lines denote the fitting results. (**a**) The effect of the inclination angle, (**b**) The effect of the temperature.

By changing the inclination angle from $-2^{\circ }$ to $-2^{\circ }$ in steps of $0.08^{\circ }$, the environmental temperature from 240 K to 360 K with a step size of 10 K, and the displacement of the *x* direction from 0μm to 30μm with steps of 2μm, we can calculate the restoring force with different parameters. Figure 6 shows the relation between the restoring force and the displacement corresponding to different inclination angles and the environmental temperature, indicating that the variation is nonlinear. For convenience, we fit the restoring force cubically as

(15) $$F_{re}=Kx+\Gamma x^{3}$$

**Figure 7:**
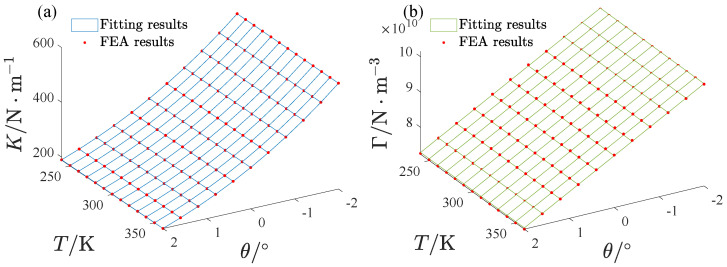
The inclination effect on the linear and nonlinear stiffness: the FEA results and fitting results. (**a**) The inclination effect on the linear stiffness, (**b**) The inclination effect on the nonlinear stiffness.

The fitting results are also shown in Figure 6. Figure 7 gives the influence of the inclination angle and the environmental temperature on *K* and Γ. We also fit them as polynomials about θ and *T*.

(16) $$\begin{array}{c} K=\left(239.256777-0.014240T\right)\left(-0.002240\theta^{3}+0.045442\theta^{2}-0.403463\theta +1.449929\right)\\ \Gamma =(3470048.686995-58.727757T)(-2072.612565\theta +25236.871261) \end{array}$$

It was demonstrated that the influence of the inclination angle on the linear stiffness is cubic, and the effect on the nonlinear stiffness is linear. The effect of the environmental temperature is very small, so the room temperature (T=300 K) was used for this study, except in Section 4.3.

### 2.4. The Static Pull-In Analysis in the Driving Direction

By setting $X=x/x_{0},Y=y/x_{0},\tau =\omega_{x}t$, Equation (7) can be rewritten in the non-dimensional form as

(17) $$\begin{array}{c} X^{\prime \prime }+X+\gamma X^{3}+\xi X^{\prime }=F_{EX}\\ Y^{\prime \prime }+GX^{\prime }+Y+\gamma Y^{3}+\xi Y^{\prime }=F_{EY} \end{array}$$

where

(18) $${\omega_{x}}^{2}=\frac{K}{M},\gamma =\frac{\Gamma {x_{0}}^{2}}{K},G=\frac{2m_{p}\omega_{z}}{M\omega_{x}},\xi =\frac{c}{M\omega_{x}}$$

The non-dimensional electrostatic forces are

(19) $$\begin{array}{c} F_{EX}=4c_{4}\beta E\cos\omega \;t+c_{1}E\left(f\left(X\right){\left(1+\beta \cos\omega \;t\right)}^{2}-\;f\left(-X\right){\left(1-\beta \cos\omega \;t\right)}^{2}\right)\;\\ +c_{7}E\left(\frac{{\left(1+\beta \cos\omega \;t\right)}^{2}}{{\left(1-X\right)}^{2}}-\frac{{\left(1-\beta \cos\omega \;t\right)}^{2}}{{\left(1+X\right)}^{2}}\right),\\ F_{EY}=c_{1}E\left(f(Y)-\;f(-Y)\right)\;+c_{7}E\left(\frac{1}{{\left(1-Y\right)}^{2}}-\frac{1}{{\left(1+Y\right)}^{2}}\right) \end{array}$$

where

(20) $$\begin{array}{c} f\left(X\right)=\frac{1+\frac{1}{c_{5}+c_{6}X}}{c_{5}+c_{6}X+\ln\left(c_{5}+c_{6}X\right)},E=\frac{N\eta {V_{D}}^{2}}{2M{\omega_{x}}^{2}x_{0}},c_{5}=1+c_{2}l,\\ c_{6}=c_{2}x_{0},c_{7}=\frac{c_{3}}{{x_{0}}^{2}} \end{array}$$

The amplitude in the driving direction is large, which can easily induce pull-in. Hence, the static pull-in analysis of the driving direction should be conducted. We consider a conservative system in the driving direction as follows.

(21) $$X^{\prime \prime }+X+\gamma X^{3}=c_{1}E\left(f(X)-\;f(-X)\right)+c_{7}E\left(\frac{1}{{\left(1-X\right)}^{2}}-\frac{1}{{\left(1+X\right)}^{2}}\right)$$

The system’s potential energy is obtained by setting the equilibrium position as the point with zero potential energy.

(22) $$\begin{array}{c} V_{C}=\frac{1}{2}X^{2}+\frac{1}{4}\gamma X^{4}-\frac{c_{1}E}{c_{6}}\{\ln\left[c_{5}+c_{6}X+\ln\left(c_{5}+c_{6}X\right)\right]\\ +\ln\left[c_{5}-c_{6}X+\ln\left(c_{5}-c_{6}X\right)\right]\}+\frac{c_{7}E}{1-X}+\frac{c_{7}E}{1+X} \end{array}$$

**Figure 8:**
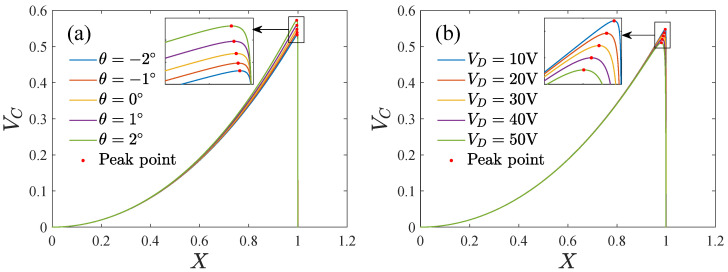
The potential energy in the driving direction, (**a**) The potential energy corresponding to different inclination angles, (**b**) The potential energy corresponding to different DC voltages.

Figure 8 shows the potential energy corresponding to different DC voltages and inclination angles. The parameter values are chosen in Table 1 in the following calculations if there are no special instructions. The system may have a stable periodic motion in the potential well near the equilibrium position X=0. Pull-in is triggered when the proof mass displacement crosses the potential barrier near X=1. The barrier height increases with the inclination angle but decreases with the DC voltage. The displacement of the peak point of the barrier can be regarded as the boundary amplitude of the pull-in.

**Figure 9:**
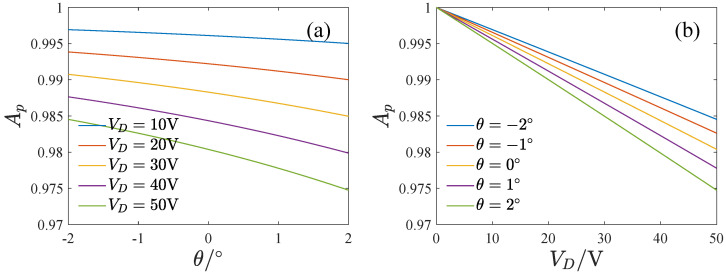
The influence of the DC voltage and the inclination angle on the pull-in boundary amplitude. (**a**) The influence of the DC voltage, (**b**) The influence of the inclination angle.

We investigated the effect of the DC voltage and the inclination angle on the pull-in boundary amplitude, as shown in Figure 9. It was found that the pull-in amplitude decreases with the DC voltage but increases with the inclination angle, which means pull-in is more likely to occur at a large DC voltage or a small inclination angle. Physically, the electrostatic force in the driving direction that causes the system to pull in increases with the DC voltage and decreases with the inclination angle. However, reducing the inclination angle leads to a rapid increase in the stiffness and the restoring force, which increases the pull-in boundary amplitude. Ignoring the inclination effect on the stiffness may bring about an opposite result. Therefore, it is necessary to consider the inclination effect on both the electrostatic forces and the stiffness.

## 3. The Periodic Solution and Its Stability of the Gyroscope

The periodic response is significant for MEMS gyroscopes. In this section, we investigate the periodic response of the gyroscope and its stability. Note that f(x) contains logarithmic terms that cannot be calculated analytically. f(x) is expanded into the Taylor series as follows.

(23) $$f\approx b_{0}+b_{1}X+b_{2}X^{2}+b_{3}X^{3}+b_{4}X^{4}+b_{5}X^{5}+b_{6}X^{6}$$

**Figure 10:**
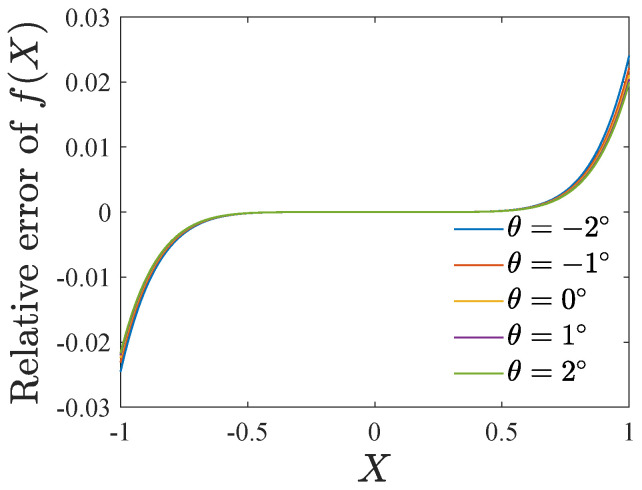
Relative error of Taylor expansion of f(X).

Figure 10 gives the relative error of the Taylor expansion of f(X). The maximum error is less than 3%. Substituting Equation (23) into Equation (19) leads to

(24) $$\begin{array}{c} F_{EXT}=4c_{4}\beta E\cos\Omega \tau +4c_{1}\beta E\left(b_{0}+b_{2}X^{2}+b_{4}X^{4}+b_{6}X^{6}\right)\cos\Omega \tau \\ +2c_{1}E\left(b_{1}X+b_{3}X^{3}+b_{5}X^{5}\right)\left(1+\beta^{2}\cos^{2}\Omega \tau \right)\\ +\frac{c_{7}E{\left(1+\beta \cos\Omega \tau \right)}^{2}}{{\left(1-X\right)}^{2}}-\frac{c_{7}E{\left(1-\beta \cos\Omega \tau \right)}^{2}}{{\left(1+X\right)}^{2}},\\ F_{EYT}=2c_{1}E\left(b_{1}Y+b_{3}Y^{3}+b_{5}Y^{5}\right)\;+\frac{c_{7}E}{{\left(1-Y\right)}^{2}}-\frac{c_{7}E}{{\left(1+Y\right)}^{2}}, \end{array}$$

The solutions to Equation (17) can be assumed as

(25) $$\begin{array}{c} X=A_{1}\cos\left(\Omega \tau +\theta_{1}\right)\\ Y=A_{2}\cos\left(\Omega \tau +\theta_{2}\right) \end{array}$$

where $A_{1},A_{2},\theta_{1},\mathrm{and}\;\theta_{2}$ are the amplitudes and phase angles of *X* and *Y*. Considering the primary resonance yields $\Omega^{2}=1+\sigma$, where σ is the detuning parameter. According to the method of averaging [18], we have

(26) $$\begin{array}{c} {A_{1}}^{\prime }=-\frac{1}{2\pi \Omega }\int_{0}^{2\pi /\Omega }(F_{EXT}+\sigma X-\gamma X^{3}-\xi X^{\prime })\sin\Omega \tau d\tau \\ A_{1}{\theta_{1}}^{\prime }=-\frac{1}{2\pi \Omega }\int_{0}^{2\pi /\Omega }(F_{EXT}+\sigma X-\gamma X^{3}-\xi X^{\prime })\cos\Omega \tau d\tau \\ {A_{2}}^{\prime }=-\frac{1}{2\pi \Omega }\int_{0}^{2\pi /\Omega }(F_{EYT}+\sigma Y-\gamma Y^{3}-\xi Y^{\prime }-GX^{\prime })\sin\Omega \tau d\tau \\ A_{2}{\theta_{2}}^{\prime }=-\frac{1}{2\pi \Omega }\int_{0}^{2\pi /\Omega }(F_{EYT}+\sigma Y-\gamma Y^{3}-\xi Y^{\prime }-GX^{\prime })\cos\Omega \tau d\tau \end{array}$$

The integrals in Equation (26) can be solved as

(27) $$\begin{array}{c} {A_{1}}^{\prime }=-\;\frac{\begin{array}{c} c_{1}E\beta^{2}\left(5b_{5}{A_{1}}^{5}+16b_{1}A_{1}+8b_{3}{A_{1}}^{3}\right)\sin\theta_{1}\cos\theta_{1}+16\xi A_{1}\Omega \\ +E\beta \left(8b_{4}c_{1}{A_{1}}^{4}+5b_{6}c_{1}{A_{1}}^{6}+16b_{2}c_{1}{A_{1}}^{2}+64b_{0}c_{1}+64\;c_{4}\right)\sin\theta_{1} \end{array}}{32\Omega }+f_{X11}\\ A_{1}{\theta_{1}}^{\prime }=-\frac{\begin{array}{c} c_{1}E\beta^{2}\left(30\;b_{5}\;c_{1}{A_{1}}^{5}+32b_{3}c_{1}{A_{1}}^{3}+32b_{1}A_{1}\right)\cos^{2}\theta_{1}-24\gamma {A_{1}}^{3}+32\sigma A_{1}\\ +E\beta \left(70b_{6}c_{1}{A_{1}}^{6}+80b_{4}c_{1}{A_{1}}^{4}+96b_{2}c_{1}{A_{1}}^{2}+128\;b_{0}c_{1}+128c_{4}\right)\cos\theta_{1}\\ +Ec_{1}A_{1}\left[\beta^{2}\left(5\;b_{5}{A_{1}}^{4}+8b_{3}{A_{1}}^{2}+16b_{1}\right)+40b_{5}{A_{1}}^{4}+48b_{3}{A_{1}}^{2}+64b_{1}\right] \end{array}}{64\;\Omega }+f_{X12}\\ {A_{2}}^{\prime }=\frac{-A_{1}G\cos\left(\theta_{1}-\theta_{2}\right)-A_{2}\xi }{2}\\ A_{2}{\theta_{2}}^{\prime }=-\;\frac{5Eb_{5}c_{1}{A_{2}}^{5}+6Eb_{3}c_{1}{A_{2}}^{3}+8Eb_{1}c_{1}A_{2}+4\;G\Omega \;A_{1}\sin\left(\theta_{1}-\theta_{2}\right)-3\gamma {A_{2}}^{3}+4\sigma A_{2}}{8\Omega }+f_{Y12} \end{array}$$

where the integral of the fractional term in $F_{EXT},F_{EYT}$ can be obtained by the residue theorem [9,19], and the expressions of $F_{X11},F_{X12}$, and $F_{Y12}$ are as shown below.

(28) $$\begin{array}{c} F_{X11}=\frac{4E\beta \;c_{7}\;\left(\begin{array}{c} \left(A_{1}-{A_{1}}^{2}\beta \cos\theta_{1}+2\;\beta \cos\theta_{1}\right)\sqrt{1-{A_{1}}^{2}}+2\;{A_{1}}^{2}\beta \cos\theta_{1}\\ +{A_{1}}^{3}-2\beta \cos\theta_{1}-A_{1} \end{array}\right)\sin\theta_{1}}{\Omega \;{A_{1}}^{3}\left({A_{1}}^{2}-1\right)}\\ F_{X12}=-\frac{2c_{7}E\;\left(\begin{array}{c} \beta^{2}\left[6{A_{1}}^{2}-{A_{1}}^{4}-4\;\right)\sqrt{1-{A_{1}}^{2}}+4{A_{1}}^{4}-8{A_{1}}^{2}+4\;]\cos^{2}\theta_{1}\\ +\beta \left[2{A_{1}}^{5}-4{A_{1}}^{3}+2A_{1}+\left(4{A_{1}}^{3}-2A_{1}\right)\sqrt{1-{A_{1}}^{2}}\right]\cos\theta_{1}\\ +\beta^{2}\left[\left({A_{1}}^{4}-3{A_{1}}^{2}+2\;\right)\sqrt{1-{A_{1}}^{2}}-2{A_{1}}^{4}+4{A_{1}}^{2}-2\right]+{A_{1}}^{4}\sqrt{1-{A_{1}}^{2}} \end{array}\right)}{\Omega \;{A_{1}}^{3}{\left(1-{A_{1}}^{2}\right)}^{2}}\\ F_{Y12}=-\;\frac{2c_{7}EA_{2}\sqrt{1-{A_{1}}^{2}}}{{\left(1-{A_{1}}^{2}\right)}^{2}\Omega } \end{array}$$

By setting ${A_{1}}^{\prime }={A_{2}}^{\prime }={\theta_{1}}^{\prime }={\theta_{2}}^{\prime }=0$, the nonlinear equations about the steady-state solutions to the periodic response can be obtained. The amplitudes and phase angles can be calculated using the homotopy continuation method. The stability of the steady-state solutions can be determined by the eigenvalues of the Jacobian matrix of Equation (27) [18].

**Figure 11:**
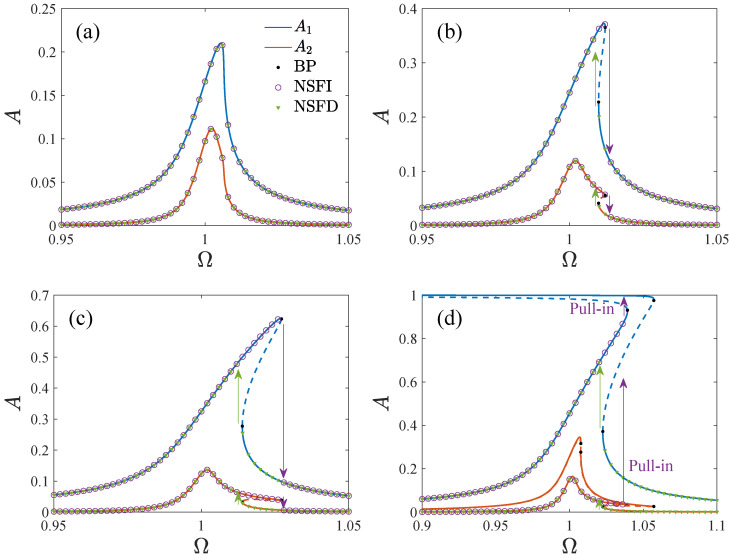
The amplitude–frequency curve versus different parameters, where the solid line is the stable solution, the dashed line is the unstable solution, BP denotes the bifurcation point, NSFI is the numerical solution as the frequency is increased, NSFD is the numerical solution as the frequency is decreased, (**a**) $V_{D}=20\;V,V_{A}=5\;V,\theta =2^{\circ }$, (**b**) $V_{D}=20\;V,V_{A}=10\;V,\theta =0$, (**c**) $V_{D}=10\;V,V_{A}=30\;V,\theta =-1^{\circ }$, (**d**) $V_{D}=20\;V,V_{A}=20\;V,\mathrm{and}\;\theta =-2^{\circ }$.

Figure 11 shows the amplitude–frequency curves of the system. By using the fourth-order Runge–Kutta method, the solutions of Equations (18) and (20) are also given to verify the analytical solutions. The results are in good agreement. Note that the amplitude–frequency curves exhibit different dynamic characteristics. In Figure 11a, $A_{1}$ and $A_{2}$ have unique solutions corresponding to any value of Ω. In Figure 11b, a jump happens with the change in Ω, while the jump leads to pull-in in Figure 11c. Thus, it is necessary to analyze the effect of the parameters on the bifurcation characteristics.

## 4. The Influence of Parameters on the Response of the Gyroscope

### 4.1. The Singularity Analysis in the Driving Direction

In this section, the singularity theory is employed to study the influence of the parameters on the bifurcation characteristics in the driving direction. It is impossible to eliminate $\theta_{1}$ to obtain the bifurcation equation about $A_{1}$ from Equation (27). Thus, the two-variable singularity theory is used in this section. By setting $A_{1}=u,\theta_{1}=v,\sigma =\lambda$, the following bifurcation equations are considered.

(29) $$G_{1}={A_{1}}^{\prime }=0,G_{2}={\theta_{1}}^{\prime }=0$$

The transition sets of Equation (29) are defined as D=B∪H∪DL, where B, H, and DL denote the bifurcation set, the hysteresis set, and the double limit point set, respectively. Their expressions are given as follows [20,21].

Bifurcation set:

(30) $$B=\left\{\left(V_{D},V_{A}\right)\in R^{2}\left|\begin{array}{c} \exists \left(u,v,\lambda \right)\;s.t.\;G_{1}=0,G_{2}=0,G_{1u}G_{2v}-G_{1v}G_{2u}=0,\\ G_{1u}G_{2\lambda }-G_{1\lambda }G_{2u}=0 \end{array}\right.\right\}$$

Hysteresis set:

(31) $$H=\left\{\left(V_{D},V_{A}\right)\in R^{2}\left|\begin{array}{c} \exists \left(u,v,\lambda \right)\;s.t.\;G_{1}=0,G_{2}=0,G_{1u}G_{2v}-G_{1v}G_{2u}=0,\\ G_{1u}\dot{u}-G_{2u}\dot{v}s.=0,G_{1u}f_{2}-G_{2u}f_{1}=0,\\ f_{1}=G_{1uu}{\dot{u}}^{2}+2G_{1uv}\dot{u}\dot{v}s.+G_{1vv}{\dot{v}}^{2},\\ f_{2}=G_{2uu}{\dot{u}}^{2}+2G_{2uv}\dot{u}\dot{v}s.+G_{2vv}{\dot{v}}^{2} \end{array}\right.\right\}$$

Double limit set:

(32) $$\mathrm{DL}=\left\{\left(V_{D},V_{A}\right)\in R^{2}\left|\begin{array}{c} \exists \left(Z_{1},Z_{2},\lambda \right)\;s.t.\;G_{1}=0,G_{2}=0,G_{1u}G_{2v}-G_{1v}G_{2u}=0,\\ Z=\left(u,v\right),Z_{1}\neq Z_{2} \end{array}\right.\right\}$$

**Figure 12:**
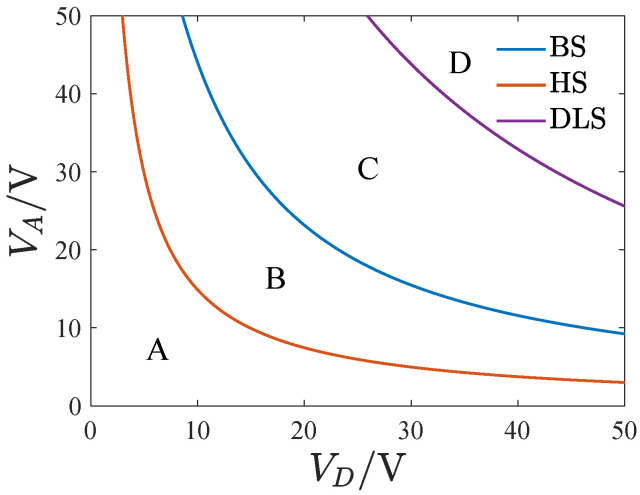
The transition sets on the DC-AC voltage plane with θ=0, where BS denotes the bifurcation set, HS denotes the hysteresis set, and DLS is the double limit point set.

**Figure 13:**
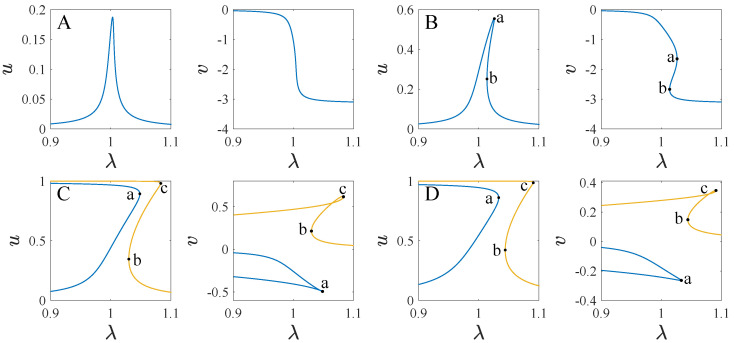
The projections of the bifurcation diagrams corresponding to different persistent regions in Figure 12.

Figure 12 shows the transition sets of the system at θ=0. The transition sets divide the parameter plane into four persistent regions. Figure 13 shows the projections of the bifurcation curves corresponding to different parameter regions on the planes of λ−u and λ−v. To illustrate the difference between the bifurcation curves, we define three key points as a, b, and c, which are the turning points of the curves. As can be seen, the system has one solution curve corresponding to the corresponding parameters in regions A and B. A single solution for u and v corresponds to any value of λ in region A, while in region B, between points a and b, u and v have three solutions. The system has two solution curves corresponding to the parameter regions C and D. In region C, point b is to the left of a, and there are five solutions between a and b. In region D, point b is to the right of point a, and a single solution exists between a and b.

**Figure 14:**
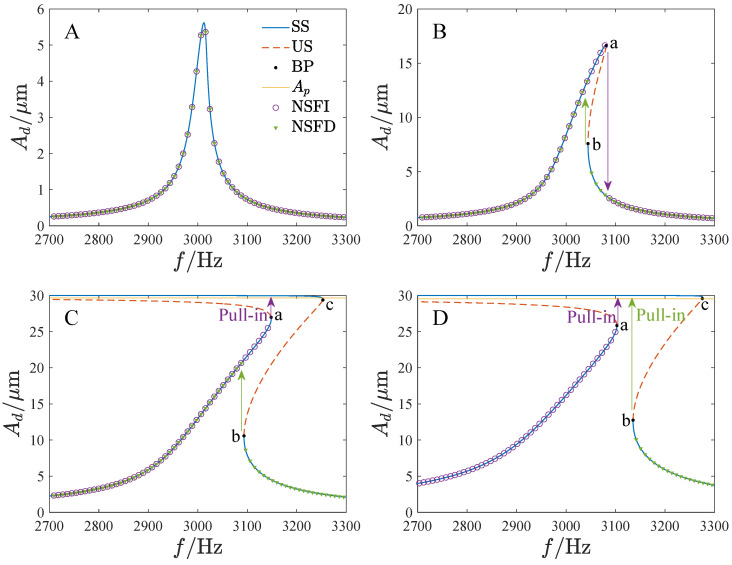
The amplitude–frequency curves in the driving direction correspond to different persistent regions in Figure 12, where SS is the stable solution, US is the unstable solution, BP denotes the bifurcation point, $A_{p}$ is the pull-in boundary amplitude, NSFI is the numerical solution as the frequency is increased, NSFD is the numerical solution as the frequency is decreased; (**A**): $V_{D}=V_{A}=10\;V$, (**B**): $V_{D}=30\;V,V_{A}=10\;V$, (**C**): $V_{D}=V_{A}=30\;V$, (**D**): $V_{D}=V_{A}=40\;V$.

Figure 14 shows the amplitude–frequency curves in the driving direction corresponding to different parameter regions. For clarity, the physical amplitudes are used instead of the non-dimensional amplitudes, where $A_{d}=A_{1}x_{0}$ and $A_{s}=A_{2}x_{0}$. The numerical results of the Runge–Kutta method are also given. They indicate that the jump phenomenon does not occur in region A. For region B, the response jumps down at point a as the frequency increases and jumps up at point b when the frequency decreases. In region C, the response pulls in after it jumps up with the frequency increase. Furthermore, the response jumps to another solution curve when the frequency is reduced. In region D, the jump of the response at both the frequency’s increasing and its decreasing leads to pull-in. The open-loop gyroscope cannot work stably in multi-solution regions because it may result in an incorrect output as the system is disturbed. Note that the peak parts of the amplitude–frequency curves of regions C and D are included in the multi-solution region. Furthermore, pull-in may occur with the change in the frequency. Thus, only regions A and B are feasible for the gyroscope operation.

**Figure 15:**
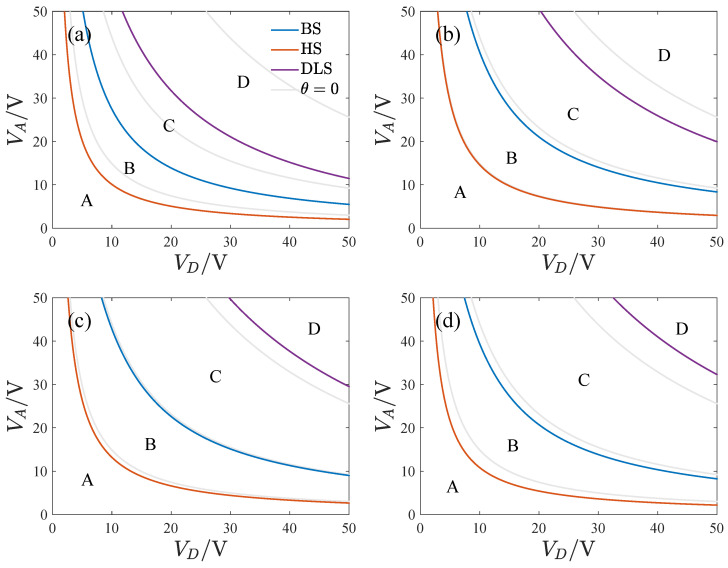
The transition sets corresponding to different inclination angles, (**a**) $\theta =-2^{\circ }$, (**b**) $\theta =-1^{\circ }$, (**c**) $\theta =1^{\circ }$, (**d**) $\theta =2^{\circ }$.

Figure 15 presents the transition sets on the $V_{D}-V_{A}$ plane corresponding to different inclination angles. Compared with θ=0, both the bifurcation set and the hysteresis set shift to the bottom left at $\theta =1^{\circ }$ and $2^{\circ }$. The gyroscope’s feasible region decreases with the inclination angle’s magnitude. As the inclination angle is increased, the double limit point set gradually shifts to the top right.

### 4.2. The Influence of Parameters on the Response of the System

**Figure 16:**
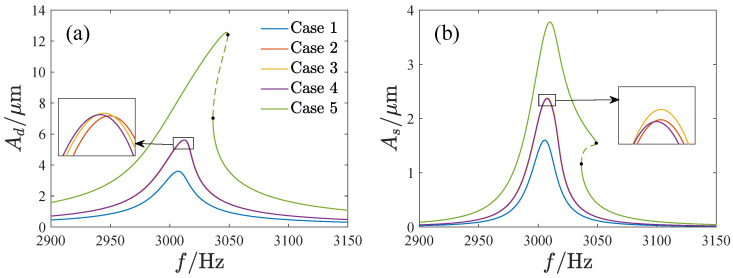
The amplitude–frequency curves corresponding to different DC and AC voltages, Case 1 $V_{D}=V_{A}=8\;V$, Case 2 $V_{D}=V_{A}=10\;V$, Case 3 $V_{D}=5\;V=V_{A}=20\;V$, Case 4 $V_{D}=20\;V=V_{A}=5\;V$, Case 5 $V_{D}=V_{A}=15\;V$. (**a**) The amplitude–frequency curves of the driving direction, (**b**) The amplitude–frequency curves of the sensing direction.

**Figure 17:**
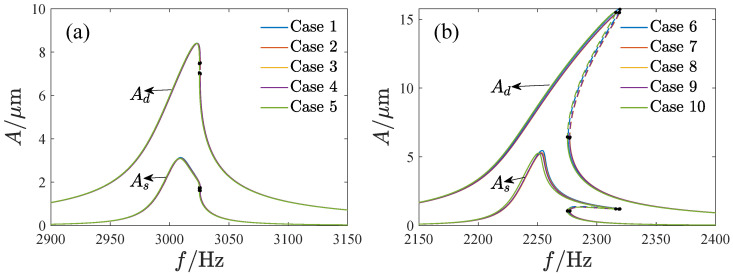
The amplitude–frequency curves corresponding to different values of $V_{D}V_{A}$ and inclination angle, (**a**) $V_{D}V_{A}$ = 150 V${}^{2}$, θ=0, (**b**) $V_{D}V_{A}$ = 250 V${}^{2}$, $\theta =2^{\circ }$.

The amplitude of the response is meaningful for the operation of the gyroscope. This section focuses on the effect of the parameters in the feasible region (regions A and B) on the amplitude of the response. Figure 16 presents the amplitude–frequency curves corresponding to different DC and AC voltages. We notice that the amplitude–frequency curves of cases 2 to 4 are almost the same. Since there is $V_{D}V_{A}=100V^{2}$ corresponding to these three cases, we suppose that the product of $V_{D}$ and $V_{A}$ is the main factor affecting the response. To verify the conjecture, Figure 17 gives the amplitude–frequency curves with different values of $V_{D}V_{A}$. The corresponding parameters are shown in Table 2. As can be seen, the amplitude–frequency curves are approximate, corresponding to the same inclination angle and $V_{D}V_{A}$ values. Therefore, $V_{D}=V_{A}=V_{E}$ is used instead of other cases with the same product of $V_{A}$ and $V_{D}$ in the following.

**Figure 18:**
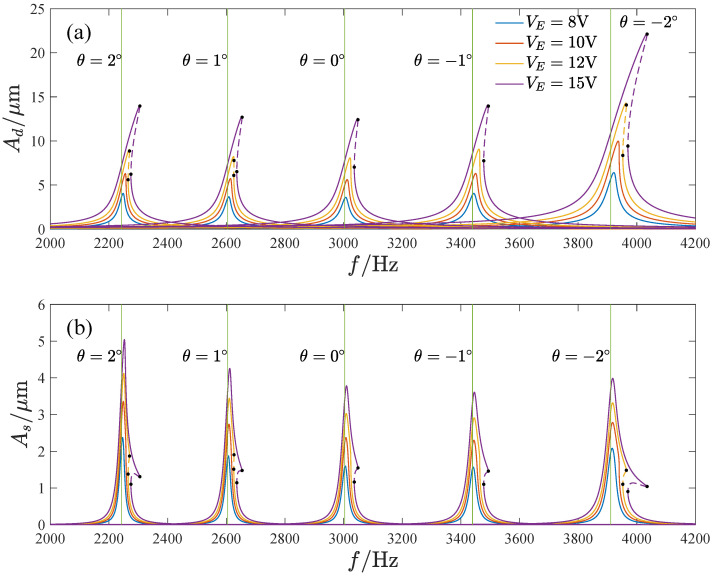
The amplitude–frequency curves corresponding to different voltage and inclination angles, (**a**) The amplitude-frequency curves in the driving direction, (**b**) The amplitude-frequency curves in the sensing direction.

Figure 18 shows the amplitude–frequency curves corresponding to different voltages and inclination angles. It can be seen that as the voltage $V_{E}$ is increased, the response amplitude increases and exhibits a stronger hardening characteristic. The natural frequency of the gyroscope decreases with the inclination angle due to the reduction in the size of the cross-section of the elastic beams as well as the bending stiffness. The amplitude of the response curves decreases first and then increases with the inclination angle. The maximum amplitude in the driving direction occurs at $\theta =-2^{\circ }$, while the maximum amplitude in the sensitive direction appears at $\theta =2^{\circ }$.

**Figure 19:**
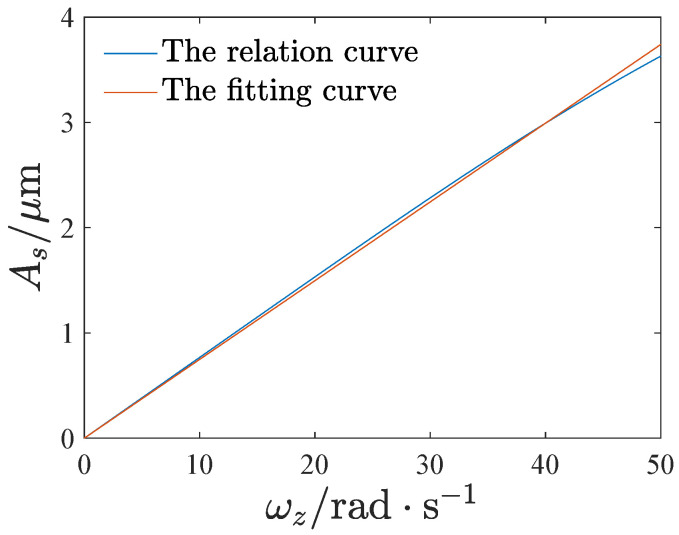
The least-squares fitting of the relation curve of the sensing amplitude and the angular velocity, $V_{E}=15\;V,\theta =1^{\circ }$.

We are also concerned about the mechanical sensitivity and nonlinearity of the gyroscope. These can be obtained from the linear least-squares fitting of the relation curve of the sensing amplitude and the angular velocity [9,22], as shown in Figure 19. We set the operating range of the gyroscope as $\omega_{z}=$0 to 50rad/s. The fitting curve is given as

(33) $$A_{sl}=S\omega_{z}$$

where *S* denotes the mechanical sensitivity. The nonlinearity can be given as

(34) $$\gamma =\frac{\max\left|A_{s}-A_{sl}\right|}{\max A_{sl}}$$

**Figure 20:**
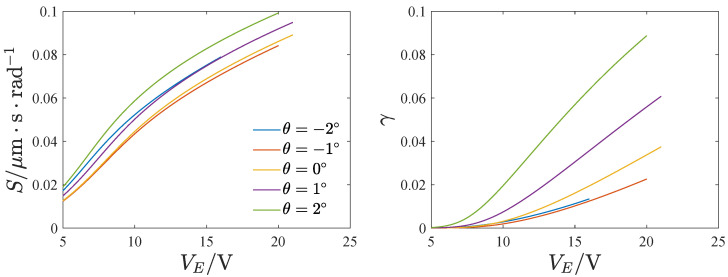
The influence of the voltage on the mechanical sensitivity and nonlinearity; Ω=1.

**Figure 21:**
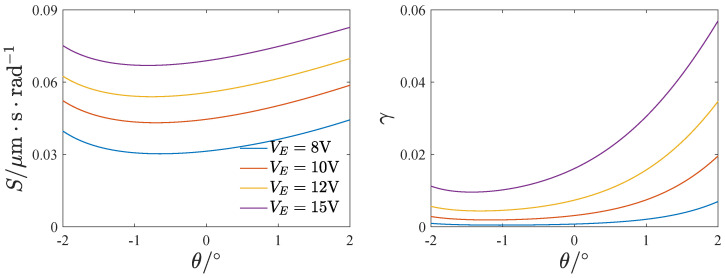
The influence of the inclination angle on the mechanical sensitivity and nonlinearity; Ω=1.

Figure 20 and Figure 21 show the effect of the voltage and inclination angle on the mechanical sensitivity and nonlinearity. Both the mechanical sensitivity and nonlinearity increase with the voltage $V_{E}$. Furthermore, as the inclination angle increases, the mechanical sensitivity and nonlinearity decrease first and then increase. A negative inclination angle leads to less nonlinearity than a positive inclination angle.

### 4.3. The Effect of the Environmental Temperature on the Mechanical Sensitivity

**Figure 22:**
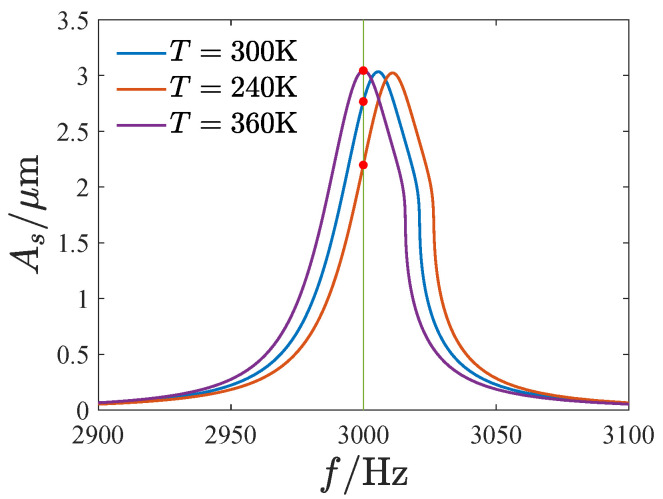
The response curves in sensing direction gyroscope corresponding to different environmental temperatures.

The change in environmental temperature may affect the output of the gyroscope. Figure 22 gives the response curve of the sensing direction corresponding to different environmental temperatures. We also set the linear natural frequency of 300 K as the working frequency. The temperature has a very small effect on the response curve, and the variation in the peak frequency of the curves is less than 1%. However, the temperature significantly affects the output amplitude of the gyroscope (shown by the red dot in Figure 22). Compared with the output of the room temperature (300 K), the output amplitude increased by 10% at 360 K and decreased by 21.5% at 240 K.

**Figure 23:**
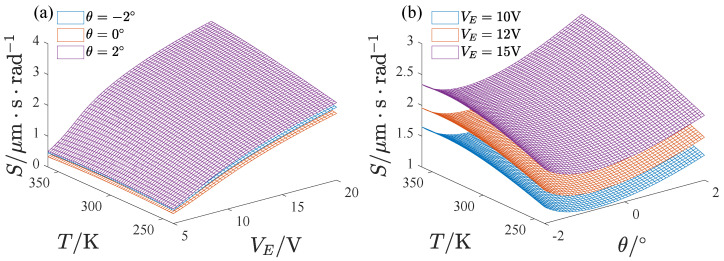
The variation in mechanical sensitivity with environmental temperature, (**a**) The effect of the voltage, (**b**) The effect of the inclination angle.

**Figure 24:**
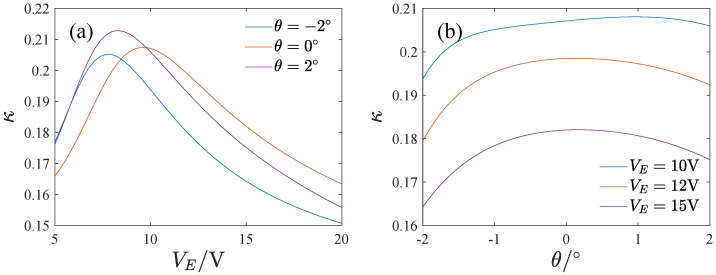
The full temperature stability of the mechanical sensitivity, (**a**) The effect of the voltage, (**b**) The effect of the inclination angle.

Figure 23 presents the variation in the mechanical sensitivity of the gyroscope in the whole temperature range. To quantify the full temperature stability of the mechanical sensitivity of the gyroscope, we define

(35) $$\kappa =\frac{\max\left|S_{T}-S_{300}\right|}{S_{300}}$$

where $S_{T}$ denotes the mechanical sensitivity of the full temperature range of the gyroscope and $S_{300}$ is the mechanical sensitivity at T=300K. Figure 24 gives the effect of the inclination angle and voltage on the full temperature stability of the mechanical sensitivity. κ increases at first and then decreases with the voltage and the inclination angle. The mechanical sensitivity changes are smaller in the whole temperature range when the voltage is large or the inclination angle is negative.

## 5. Conclusions

This paper studies the nonlinear dynamics of a symmetric MEMS gyroscope. The inclination effects on both the support stiffness and electrostatic forces are considered. The dynamic equation is established using the Lagrangian equation. The linear and nonlinear stiffness varied with the inclination angle, and environmental temperature is studied by finite element analysis. Furthermore, a static pull-in analysis in the driving direction was performed. It was found that the influence of the inclination angle on the linear stiffness is cubic, and the effect on the nonlinear stiffness is linear. The pull-in boundary amplitude decreases with the inclination angle. Ignoring the inclination effect on the stiffness leads to the opposite result.

The steady-state periodic response was obtained using the averaging method. The two-variable singularity theory was employed to study the influence of parameters on the bifurcation characteristics in the driving direction and gave the transition sets on the DC-AC voltage plane, which divided the parameter plane into four persistent regions. The corresponding bifurcation diagrams and amplitude–frequency curves are presented. The effect of the inclination angle on the transition sets is analyzed. The results show that the peak part of the amplitude–frequency curve is contained in the multi-solution region corresponding to the two regions with large voltage, and the pull-in phenomenon may occur with the change in the driving frequency. Only the two parameter regions with low voltages meet the operational requirements of the gyroscope. Both over- and under-etching may lead to the narrowing of the feasible region for the gyroscope.

The effect of parameters on the amplitude–frequency curve and the mechanical sensitivity and nonlinearity are studied in the feasible region. It demonstrates that the amplitude–frequency curves with the same product of the DC and AC voltages are almost the same. The amplitude of the response, mechanical sensitivity, and nonlinearity increase with the voltages. The response shows stronger hardening characteristics with the rise in amplitude. The increase in the inclination angle significantly reduces the natural frequency of the gyroscope. The mechanical sensitivity and nonlinearity decrease first and then increase with the inclination angle. The full temperature stability of the mechanical sensitivity is also considered. The variation in mechanical sensitivity is small at a large voltage and small inclination angle. Although the mechanical sensitivity of under-etching and over-etching is approximate, under-etching is more beneficial to the performance of the gyroscope for the small nonlinearity and good temperature stability.

## Figures and Tables

**Table 1:** The parameters of the MEMS gyroscope [16].

Parameters	Values
The proof mass $m_{p}$	0.697 mg
The mass of the outer frame $m_{i}$	0.131 mg
The length of internal elastic beam $L_{in}$	1660μm
The length of external elastic beam $L_{ex}$	800μm
The width of the elastic beams $w_{b}$	15μm
The dielectric constant η	8.85 pF
The structural thickness of the gyroscope *h*	80 μm
The separation of the combs $x_{0}$	30 μm
The finger spacing *d*	4 μm
The width of the fingers *w*	12 μm
The length of the fingers $L_{b}$	80 μm
The overlap length of the fingers *L*	50 μm
The number of fingers on a single side *N*	200
The quality factor *Q*	116.95
The DC voltage $V_{D}$	30V
The amplitude of the AC voltage $V_{A}$	10 V
The angular velocity being measured $\omega_{z}$	50 rad/s
The environmental temperature *T*	300 K
The inclination angle θ	0

**Table 2:** The parameters corresponding to different cases in Figure 16.

Case	$V_{D}$/V	$V_{A}$/V	$\theta {/}^{\circ }$	Case	$V_{D}$/V	$V_{A}$/V	$\theta {/}^{\circ }$
1	5	30	0	6	5	50	2
2	10	15	0	7	10	25	2
3	$\sqrt{150}$	$\sqrt{150}$	0	8	$\sqrt{250}$	$\sqrt{250}$	2
4	15	10	0	9	25	10	2
5	30	5	0	10	50	5	2

## Data Availability

The data that support the findings of this study are available from the corresponding author (Lijuan Zhang) upon reasonable request.
